# Plasticity in climate change responses

**DOI:** 10.1111/brv.70056

**Published:** 2025-07-23

**Authors:** Angelika Stollewerk, Pavel Kratina, Arnaud Sentis, Catalina Chaparro‐Pedraza, Ellen Decaestecker, Luc De Meester, Ozge Eyice, Lynn Govaert, John Iwan Jones, Christian Laforsch, Carolina Madeira, Anita Narwani, Vicencio Oostra, Joost A. M. Raeymaekers, Axel G. Rossberg, Matthias Schott, Robby Stoks, Ellen van Velzen, David Boukal

**Affiliations:** ^1^ Department of Biology, School of Biological and Behavioural Sciences Queen Mary University of London Mile End Rd London E1 4NS UK; ^2^ INRAE, Aix Marseille Univ., UMR RECOVER Aix‐en‐Provence 13182 France; ^3^ Fish Ecology and Evolution Department Eawag Seestrasse 79 Kastanienbaum CH‐6047 Switzerland; ^4^ Aquatic Biology, IRF Life Sciences KU Leuven E. Sabbelaan 53 Kortrijk 8500 Belgium; ^5^ Leibniz Institute of Freshwater Ecology and Inland Fisheries (IGB) Müggelseedamm 310 Berlin 12587 Germany; ^6^ Laboratory of Freshwater Ecology, Evolution and Conservation KU Leuven Ch Deberiotstraat 32 Leuven 3000 Belgium; ^7^ Institute of Biology Freie Universität Berlin Köningin‐Louise‐Strasse 1‐3 Berlin 14195 Germany; ^8^ Animal Ecology and BayCEER University of Bayreuth Universitaetsstrasse 30 Bayreuth 95447 Germany; ^9^ Associate Laboratory i4HB ‐ Institute for Health and Bioeconomy, NOVA School of Science and Technology NOVA University Lisbon Caparica 2819‐516 Portugal; ^10^ UCIBIO – Applied Molecular Biosciences Unit, Department of Life Sciences, NOVA School of Science and Technology NOVA University Lisbon Caparica 2819‐516 Portugal; ^11^ Aquatic Ecology Department Eawag Überlandstrasse 133 Dübendorf CH‐8600 Switzerland; ^12^ Faculty of Biosciences and Aquaculture Nord University Bodø N‐ 8049 Norway; ^13^ Laboratory of Evolutionary Stress Ecology and Ecotoxicology KU Leuven Ch Deberiotstraat 32 Leuven 3000 Belgium; ^14^ Department of Ecology and Ecosystem Modelling University of Potsdam Am Neuen Palais 10 Potsdam 14469 Germany; ^15^ Department of Ecosystems Biology, Faculty of Science University of South Bohemia Branišovská 31a České Budějovice 370 05 Czech Republic; ^16^ Biology Centre, Institute of Entomology Czech Academy of Sciences Branišovská 31 České Budějovice 370 05 Czech Republic

**Keywords:** climate change, warming, microbiome, phenotypic plasticity, molecular pathways, species interactions, community, ecological feedbacks

## Abstract

Recent research has shown that climate change can both induce and modulate the expression of plastic traits but our understanding of the role of phenotypic plasticity as an adaptive response to climate change is limited. In this review, we dissect the mechanisms and impact of phenotypic plasticity as a response to accumulating climatic pressures on the individual, species and community levels. (*i*) We discuss how plasticity can affect individuals, populations and community dynamics and how climate change can alter the role of plasticity. We hypothesise that some pathways to phenotypic plasticity such as irreversible and anticipatory organismal responses will be reduced under increasing climate change. (*ii*) We then propose an integrated conceptual framework for studying phenotypic plasticity to advance our understanding of the feedbacks between the different levels of biological organisation. (*iii*) By formulating as yet unaddressed research questions within and across levels of biological organisation, we aim to instigate new research on phenotypic plasticity and its role in climate change responses.

## I. INTRODUCTION

Ongoing and future climate change is accompanied by unprecedented challenges for Earth's ecosystems. Terrestrial and aquatic environments have all been subjected to sudden amalgamations of threats, resulting in damaged habitats and widespread species declines (Chapin III *et al*., 2000). How do organisms cope with these rapid and often unpredictable environmental changes? Most organisms exhibit phenotypic plasticity, which we define as “the ability of an organism to react to an environmental input with a change in form, state, movement, or rate of activity” (West‐Eberhard, 2003, p. 33) and which can often compensate for environmental changes (Pigliucci, 2001; Radchuk *et al*., 2019). However, little is known about the role of phenotypic plasticity in ecological communities facing accelerating climate change. Although some progress has been made in understanding the role of phenotypic plasticity in competitive communities (Hess *et al*., 2022; Bazin *et al*., 2024), we lack a conceptual framework for evaluating the role of plasticity in complex natural communities with multiple trophic levels and different types of species interactions. Phenotypic plasticity is itself an evolving trait, exhibiting genetic variation (e.g. Mallard, Nolte & Schlotterer, 2020), and can influence the direction of evolutionary change depending on the adaptive value of the plastic trait (e.g. non‐adaptive phenotypic plasticity might lead to extinction or rapid adaptive evolution). This relationship between evolution and phenotypic plasticity has been discussed in depth elsewhere (e.g. Gibert, Debat & Ghalambor, 2019; Merilä & Hendry, 2014).

**Fig. 1:**
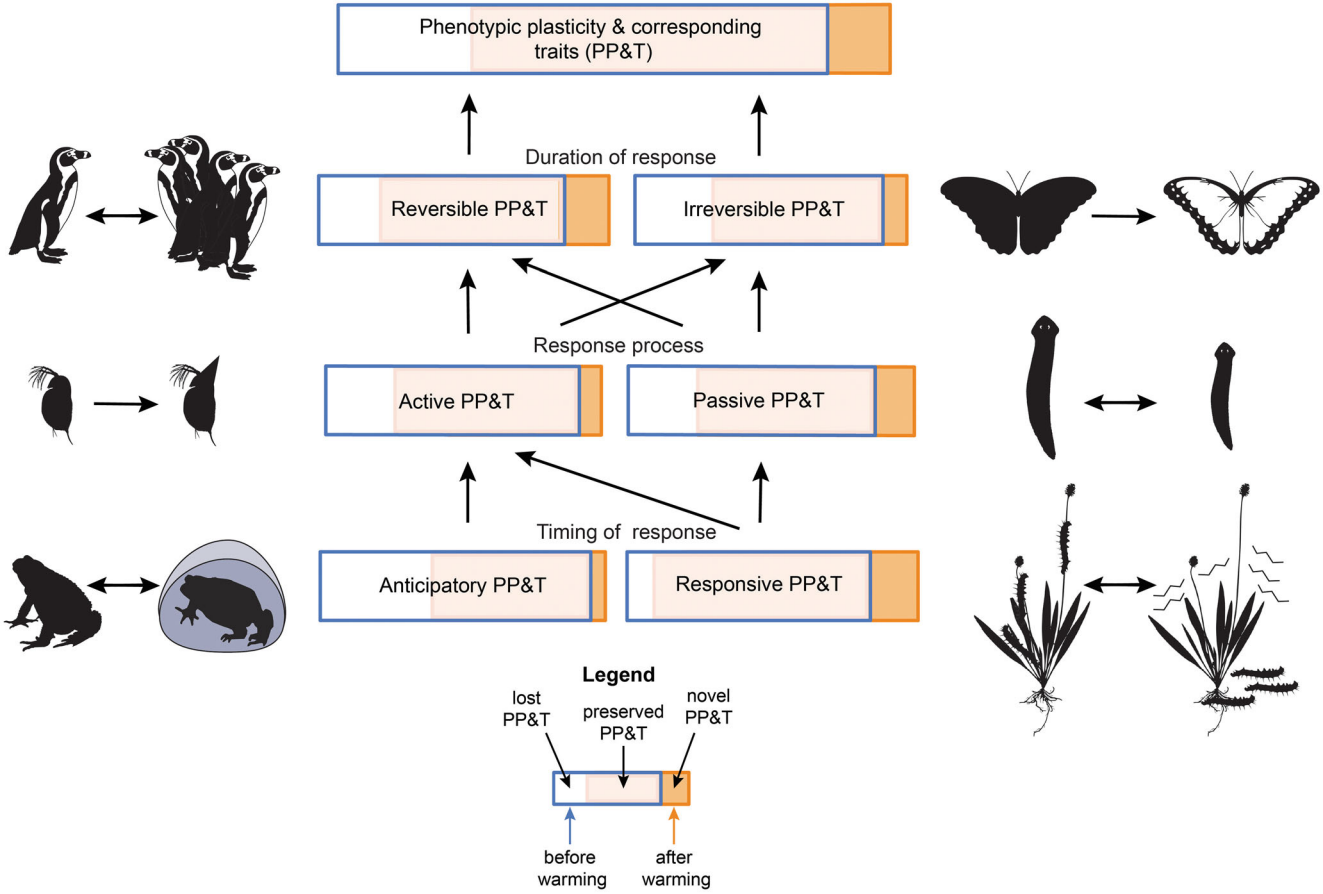
Relationships between the different aspects of phenotypic plasticity and corresponding traits (PP&T) and their possible changes caused by climate warming. Each rectangle represents the diversity of plastic traits and their quantitative expression before (blue unfilled areas) and after (orange‐filled areas) warming. The arrows between the rectangles indicate possible combinations of the different aspects of plasticity. In this hypothetical example, the overall amount of phenotypic plasticity (i.e. the number of phenotypically plastic traits and the overall magnitude of their expression, represented by the rectangle area) declines after warming (orange area is smaller) because more traits become non‐plastic and/or diminish in plasticity (lost PP&T) relative to “novel” plastic traits or increased levels of plasticity (novel PP&T) after warming. These changes and the proportion of PP&T that is not affected by warming (preserved PP&T) will likely not be uniform across the different aspects of plasticity. For example, anticipatory PP&T and irreversible PP&T can be lost significantly more than gained and the opposite may hold for responsive PP&T after warming. The silhouettes show examples of plasticity and traits that could be directly or indirectly affected by climate warming (double‐headed arrows: reversible plasticity; unidirectional arrows: irreversible plasticity). Left (from the top): reversible plasticity: huddling behaviour in penguins; active plasticity: defence formation in *Daphnia*; anticipatory plasticity: hibernation in toads. Right (from the top): irreversible plasticity: seasonal pigmentation in butterflies; passive plasticity: degrowth of planarians after resource depletion; responsive plasticity: insect larvae attack on plant followed by release of toxins (modified images from PhyloPic; planarian picture by Noah Schlottman https://creativecommons.org/licenses/by‐sa/3.0/).

Phenotypic plasticity can involve morphological (e.g. body size and shape), physiological (e.g. stoichiometric homeostasis, metabolic and excretion rates, aerobic scope), behavioural (e.g. habitat, diet selection, orientation) and life‐history traits (e.g. age, size at maturity, fecundity), as well as learning (e.g. physical and social cognition), adaptive immunity and microbiome composition, which affects the host's phenotype. For all of these trait types, there are several overlapping pathways leading to the plastic response, which are based on the timing of environmental change and the organism's response, the processes involved in the plastic response and the duration of the trait response (Fig. 1). The terms we use here follow established definitions (Whitman & Agrawal, 2009). *Anticipatory phenotypic plasticity* is a response to an environmental cue that predicts a future event (e.g. diapause triggered by photoperiod), while *responsive phenotypic plasticity* is a direct response to an environmental stimulus (e.g. the production of plant toxins following herbivore attack; Halkier & Gershenzon, 2006). While anticipatory phenotypic plasticity is always an active process, including a switch to alternative developmental pathways, for example the formation of defence structures in *Daphnia* offspring from mothers exposed to predator kairomones (Laforsch & Tollrian, 2004) or specific changes in behaviour, for example seasonal activities in great tits (*Parus major*) (Bonamour *et al*., 2019), responsive phenotypic plasticity can be subdivided into *active and passive phenotypic plasticity* (Fig. 1). Active plastic traits are generated by the activation of specific molecular pathways, whereas passive phenotypic plasticity arises through environmental stimuli acting on metabolic processes, which in turn leads to phenotypic changes. For example, slower growth is a passive response to resource depletion, whereas faster growth to escape predation risk represents an active response (Stoks, Swillen & De Block, 2012). Both types may be combined to produce the overall response to environmental change. For example, lower temperature can decrease fecundity in the ladybird *Menochilus sexmaculatus* (primary passive response) but this can be buffered by plastic melanism leading to a darker morph that is more fecund at low temperature than the regular non‐melanised morph (Dubey, Omkar & Mishra, 2016). *Developmental plasticity* is a sub‐category of phenotypic plasticity and refers to the ability of an organism to divert developmental processes along alternative pathways to generate a plastic phenotype in response to internal or external cues during ontogenesis (Gilbert & Epel, 2015). Examples include nutritional polyphenism in hymenopteran insects and seasonal variation in pigmentation in butterflies (Gilbert & Epel, 2015).

Furthermore, plastic traits can be *reversible (labile)* if they can be undone rapidly in response to the environment (Fig. 1). Examples are the enhanced phototactic behaviour of *Daphnia* in the presence of predators (Bellot, Gómez‐Canela & Barata, 2022; Van gool & Ringelberg, 1998; De Meester, 1993), the switching between navigational strategies in honeybees depending on meteorological conditions (Chittka & Geiger, 1995) or the huddling behaviour of penguins (Richter *et al*., 2018). Plastic physiological traits are also often reversible on very short timescales, such as the alteration in photosynthetic rates in plants in response to light or the production of red blood cells due to reduced oxygen levels (Pigliucci, 2001). *Irreversible (fixed)* phenotypic plasticity refers to traits that remain constant, for example, after completing a certain phase in ontogeny [e.g. maturation age and size (Whitman & Agrawal, 2009), bone structure (Campbell *et al*., 2021)]. We emphasise that behavioural and physiological plastic traits are often reversible but can also be irreversible, such as predatory feeding behaviour in nematodes (Wilecki *et al*., 2015), developmental physiological changes induced by limited nutrition in humans (Gilbert & Epel, 2015) or incubation conditions of reptilian eggs (Refsnider, Clifton & Vazquez, 2019). On the other hand, some morphological plastic traits can be reversible, such as seasonal changes in organ size in migratory birds (Piersma & Lindström, 1997; Elowe, Babbitt & Gerson, 2023) or degrowth and regrowth in planarians (Ko *et al*., 2024; Fig. 1).

**Fig. 2:**
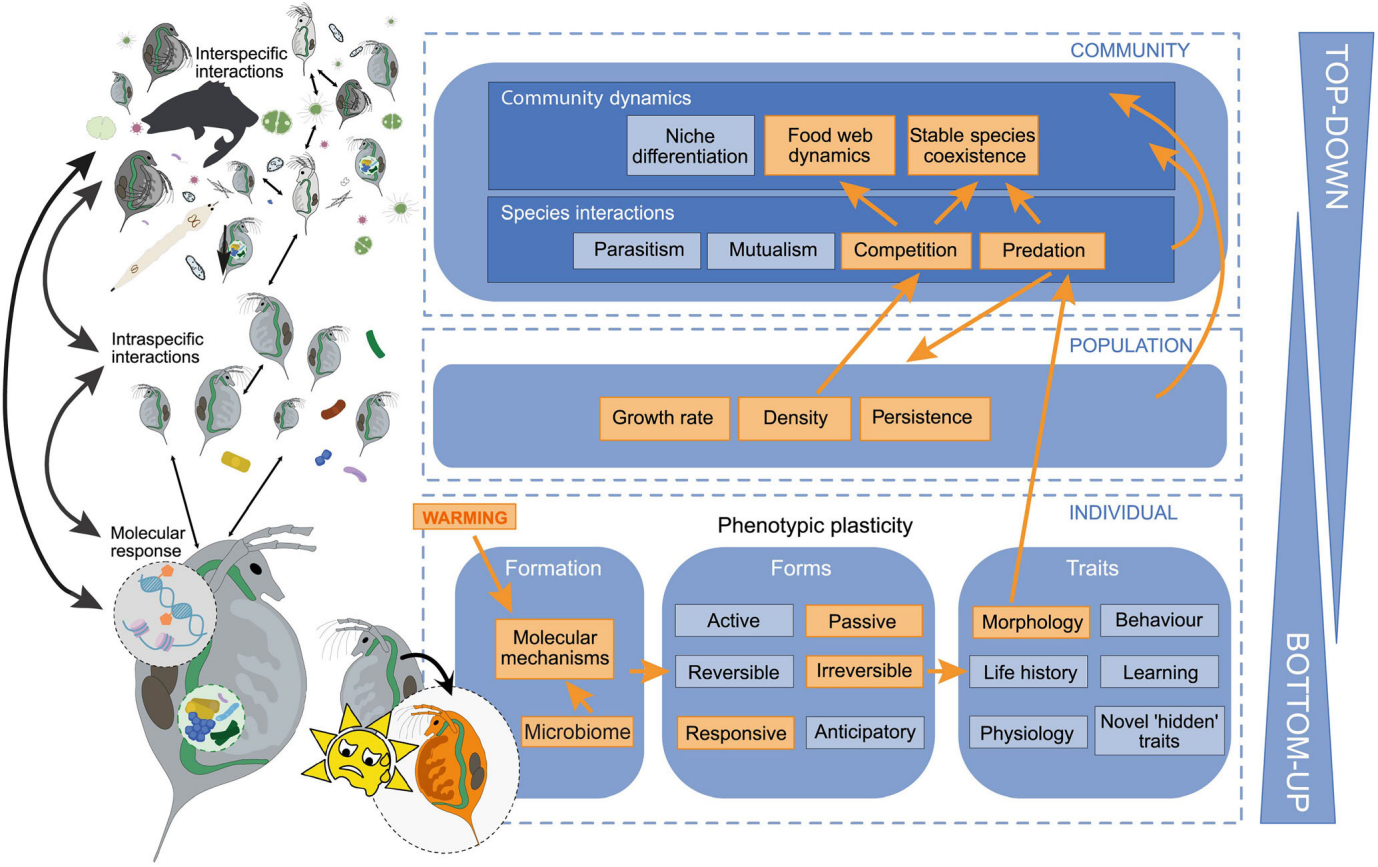
Conceptual framework integrating bottom‐up and top‐down approaches for understanding phenotypic plasticity at different levels of biological organisation. Phenotypic plasticity (curved black arrow on image on bottom row) occurs at the individual level and can be induced by abiotic environmental responses (e.g. warming illustrated by orange colours) as well as by biotic changes in population (e.g. density) and community (e.g. predation, competition, resources, parasites) state. The response to the environmental cue can be modified by the microbial community (microbiome) composition associated with the individual. Here, we highlight an example of phenotypic plasticity in response to warming, the type of plasticity response (active *versus* passive, reversible *versus* irreversible and responsive *versus* anticipatory) and the type of trait (morphology, behaviour, life history, learning, physiology or novel “hidden” traits) that can show the plastic response (orange arrows and rectangles at the individual level). Phenotypic plasticity at the individual level can have bottom‐up effects at the population and community level. For example, higher temperatures can result in a smaller body size in *Daphnia*, which can modify interactions with predatory aquatic invertebrates that prefer smaller prey (Pastorok, 1981) and consequently, for example alter prey density, with further effects on population and community level (orange arrows and rectangles connecting the individual, population and community level). Altered ecological interactions at the community or population level could further induce different phenotypic plasticity responses, studied from a top‐down approach. This illustrates the many potential ways phenotypic plasticity can result in changed interactions affecting individual‐, population‐ (e.g. intraspecific interactions, indicated by thin black double‐headed arrows on the left‐hand panel) and community‐level processes (e.g. interspecific processes, indicated by thin black double‐headed arrows on the left‐hand panel) resulting in many potential feedbacks between different levels of ecological organisation in all directions (large double‐headed arrows on left‐hand side).

Here we review the short‐ and long‐term impacts of phenotypic plasticity as an adaptive response to accumulating environmental pressures from climate change. In particular, we focus on the maintenance of community structure and dynamics in response to climate warming and associated extreme climatic events (Reed, Schindler & Waples, 2011). We consider the mechanisms underlying the development and impact of phenotypic plasticity at the individual, population and community levels to build a conceptual framework for understanding the interconnecting factors and feedback among the different levels of biological organisation. In particular, we propose to combine top‐down and bottom‐up approaches to understand better the impacts of plastic responses to climate change (Fig. 2).

A bottom‐up approach treats population and community dynamics as emergent phenomena arising from the sum of individual life histories (De Roos & Persson, 2013; Cole, 1954). This reductionist approach investigates the effects of climate change on individual‐level processes and key traits, including somatic growth rate, maturation age and body size, providing insights into the role of phenotypic plasticity at the organismal level and its consequences for the dynamics of populations and communities (Reed *et al*., 2011; Brass *et al*., 2021; Forsman, 2015; Zettlemoyer & Peterson, 2021). However, it may overlook potential feedback from the community that could further influence individual‐level processes. By contrast, a top‐down approach investigates the complexity of communities and aims to quantify the relative importance of different processes at the individual, population and community level. In so doing it can reveal the importance of interactions that might remain undetected in bottom‐up approaches (e.g. ecological and evolutionary feedbacks of species interactions and community changes on individuals). However, with a top‐down approach it is often difficult to understand the mechanisms that link phenotypic plasticity at the individual level to the phenomena at population and community levels, and their interactions. Since feedbacks can potentially occur between all levels in natural communities as a result of plastic responses, we propose that combining both approaches is necessary to understand fully the phenotypic responses of ecological communities to climate change.

Our synthesis first focuses on bottom‐up approaches, outlining the mechanisms of formation and the constraints of phenotypic plasticity at the individual level. We also consider which aspects of phenotypic plasticity may become more or less important in organismal responses to climate change (Fig. 1). We then propose the combination of bottom‐up and top‐down approaches to evaluate how the rate of thermal change, thermal variability and plasticity constraints shape the distribution of phenotypes at the community level and explore the consequences for community dynamics and persistence. Finally, considering each of these approaches, we highlight unanswered research questions on the role of phenotypic plasticity for climate change responses.

## II. BOTTOM‐UP APPROACH – EFFECTS OF CLIMATE CHANGE ON INDIVIDUALS

### (1) Climate change induces diverse context‐dependent plastic responses

Phenotypic plasticity is an integral part of the development and life cycle of most species, contributing to variations among individuals [e.g. nutrition‐induced castes in Hymenoptera (Roth *et al*., 2019); temperature‐induced pigmentation in spiders and butterflies (Blanke & Merklinger, 1982; Bhardwaj *et al*., 2020)]. Many examples show that climate change both induces and interferes with the formation of plastic traits. For example, simulated heat waves can inhibit the development of plastic phenotypes in aphids, which normally show a transgenerationally induced winged (dispersal) phenotype under predation risk from ladybird beetles (Sentis *et al*., 2018). Higher temperatures can result in a smaller body size in *Daphnia*, which modifies predator–prey interactions with predatory aquatic invertebrates that prefer smaller prey (Pastorok, 1981) (Fig. 2). Other examples show that salmonids (*Brachymystax lenok tsinlingensis*) reared at warmer winter temperature lack antipredator responses to alarm cues (Xia *et al*., 2021) and that drivers of climate change, such as greenhouse gases (CO_2_), can interfere with chemical communication within and between species (reviewed by Draper & Weissburg, 2019), resulting in the reduction of plastic behavioural responses (Boullis *et al*., 2017).

Despite the numerous examples, our knowledge of the molecular mechanisms underlying the expression of plastic traits is still limited (Table 1), hindering our ability to establish a link between changing environmental factors and phenotypic outcome. Establishing causation is further complicated by the difficulty in categorising the relationships between environmental factors and plastic phenotypes across species, since the same cue can result in entirely different phenotypes in different species. For example, crowding induces dormancy in the worm *Caenorhabditis elegans* (Cassada & Russell, 1975) but triggers the migratory or dispersal phenotype in the desert locust *Schistocerca gregaria* and in aphids (Anstey *et al*., 2009; Hazell *et al*., 2005). Similarly, the engagement of the same signalling pathway by various environmental factors can lead to the expression of diverse phenotypes in different species. In crickets, temperature changes activate the neuroendocrine system resulting in modification of the juvenile hormone titre and affecting wing length, while in termites alterations of the juvenile hormone titre result in caste differentiation (Korb & Hartfelder, 2008).

**Table 1:** Major molecular pathways activated by climate change‐relevant environmental factors. The table shows examples of the effect of the activation of the molecular pathways by temperature. Increased temperature can act as a transcriptional regulator upregulating epigenetic regulatory enzymes. It can activate the neuroendocrine system resulting in the release of hormones and the regulation of multiple traits. Temperature can directly activate cellular sensors but also indirectly activate molecular pathways *via* the microbiome, symbionts and parasites.

Molecular pathway	Effect	Examples	
		Organism	Outcome
Transcriptional regulation of epigenetic regulatory enzymes	Epigenetic modification of DNA	Guinea pigs (*Cavia aperea*); crops (e.g. *Oryza sativa*)	Thermoregulation ‐ maintenance of temperature homoeostasis (Weyrich *et al*., 2016); heat‐stress adaptation (He *et al*., 2020)
Activation of neuroendocrine system	Release of hormones, transcriptional regulation of multiple traits	Butterflies (e.g. *Bicyclus anynana*)	Adaptation to seasonal changes [e.g. wing phenotype, physiology, metabolic rate (Monteiro *et al*., 2015; van Bergen *et al*., 2017)]
Direct activation of cellular signal transduction pathways	Increase in membrane fluidity, opening of Ca^2+^ channels; activation of opsins	Wheat (*Triticum aestivum*); flies (*Drosophila melanogaster*)	Physiological adaptation to heat (Abdelrahman *et al*., 2020); behavioural adaptation (thermotaxis) in larvae (Sokabe *et al*., 2016)
Indirect activation by microbiome, symbionts, parasites	Histone modification in heat‐stress memory genes	*Enterobacter* sp. and *Arabidopsis thaliana*; *Nematostella vectensis*; dengue (DENV), *Wolbachia pipientis* and *Aedes aegypti*	Increased heat tolerance (Shekhawat *et al*., 2021; Baldassarre *et al*., 2023); decreased heat tolerance (Ware‐Gilmore *et al*., 2021)

It follows from the above that more data on the molecular processes of phenotypic plasticity formation in a wide range of species and conditions are required to understand how climate change induces different phenotypes and to elucidate if some molecular mechanisms are better suited than others to produce adaptive plastic phenotypes under climate change. For example, the neuroendocrine system can convey information about multiple environmental conditions to different tissues, a feature termed ‘hormonal pleiotropy’ (Ketterson, Atwell & McGlothlin, 2009; Ledon‐Rettig & Ragsdale, 2021; Meylan, Miles & Clobert, 2012). The response to the systemic signal is a local property of the cells, tissues or organs that produce each plastic trait (determined by, e.g. receptor activity, intracellular localisation, expression levels). This allows a local, trait‐specific, flexible response, in which multiple traits can respond differently (Ketterson *et al*., 2009) while staying tuned to the same signal (e.g. Monteiro *et al*., 2015). Under climate change, the linked regulation of susceptible traits by hormonal cascades can lead to various outcomes (Meylan *et al*., 2012). If climate change moves the optima of all traits in the same direction, hormonal integration of plastic traits may lead to a rapid adaptive shift and contribute to population persistence, as plastic, hormonally integrated traits can shift within a generation (Meylan *et al*., 2012). If the new optima shifts for some traits but not for others, a hormonally co‐regulated suite of traits might limit the potential for phenotypic changes and lead to maladaptive traits in the novel environment (Ketterson *et al*., 2009; Meylan *et al*., 2012). However, climate change can also result in the decoupling of traits. Studies on closely related butterfly species suggest that hormonally co‐regulated, temperature‐dependent seasonally plastic traits such as abdominal fat content and dorsal wing eyespot size can become uncoupled in different geographical environments (van Bergen *et al*., 2017). Since climatic stressors such as extreme heat and drought can directly influence hormone levels, decoupling of hormonally regulated plastic traits might occur within a generation (e.g. Ruthsatz *et al*., 2020). For instance, a decrease in hormone levels at higher temperature could decouple traits that are less sensitive to hormone signalling, while traits with high sensitivity remain correlated. Moreover, trait hormonal responses are often non‐linear, such as threshold responses or responses to timing of a hormonal peak (e.g. Oostra *et al*., 2011), and such non‐linear responses typically differ among traits. This further complicates predictions of how hormonally regulated suites of plastic traits may be disrupted under climate change.

Recently, an additional molecular pathway to phenotypic plasticity has come into focus that has considerable potential to shed light on our understanding of individual (and population) variations to climate change responses: microbiome‐mediated phenotypic plasticity (Table 1). Microbial communities (microbiomes) show adaptive responses to signals from the surrounding environment by their own phenotypic plasticity, rapid evolutionary adaptations (often based on shifts in relative abundance of strains), and change in diversity at the species level (Shen & Chou, 2016; Chase, Weihe & Martiny, 2021; Xiang *et al*., 2022). The microbiome therefore adds an important source of metabolic flexibility to the host (Berg *et al*., 2020) and can be considered as an additional level of the host's phenotypic plasticity in response to environmental stress, including increased temperature, because it affects the host's phenotype without changing its genotype (Decaestecker *et al*., 2024). Multiple examples show that mutualistic symbionts can confer thermal tolerance to ectothermic hosts and alter their evolutionary trajectory under stressful temperatures (Stock *et al*., 2021; Hector *et al*., 2022; Hoang, Gerardo & Morran, 2021; Baldassarre, Reitzel & Fraune, 2023). On the other hand, in cases where symbionts are heat sensitive, warming can disrupt the microbiome and lead to lower host survival (Hector *et al*., 2022; Flandroy *et al*., 2018). Thus, the benefit of microbiome‐mediated phenotypic plasticity under climate change will depend on the community composition of the microbiome.

Despite the growing interest in the role of the microbiome in host acclimatisation and adaptive responses, the mechanisms controlling microbiome assembly and host responses to stressful environmental conditions are poorly understood (Lafuente *et al*., 2023). Microbial strains can be transmitted vertically from mother to offspring but also horizontally during an individual's lifetime (Salem *et al*., 2015). Events that occur during critical periods of development shape the structure and diversity of the microbiome and can have a lasting impact on the phenotype of the host and its offspring. For example, disruption of the microbiome of the large cabbage white butterfly *Pieris brassicae* affects the ability of the next generation to adapt to a new host plant (Paniagua Voirol *et al*., 2020). Long‐term legacy effects are likely to be more relevant for vertically transmitting microbial strains than for horizontally transmitted microbiomes because they are more consistently transmitted across different generations (Bruijning *et al*., 2022). On the other hand, flexible, horizontally derived microbiomes are potentially more important for rapid, plastic responses (Macke *et al*., 2017b) because they have a greater chance of including microbiota adapted to specific environmental conditions (Macke *et al*., 2020). For example, the microbiome of *Daphnia* exposed to cyanobacterial blooms mediates tolerance to this stressor when inoculated into the gut of germ‐free juvenile *Daphnia* (Macke *et al*., 2017a) and that effect seems to be mediated by locally adapted microbiomes (Houwenhuyse *et al*., 2021). In summary, these studies demonstrate that the host microbiome plays a key role in the host's acclimation and adaptation (*via* host genotype × microbiome interactions), but additional research is needed to address the gaps in our knowledge of the mechanisms governing the adaptation process and to quantify the relative contributions of both the host and the microbiome to the overall response (Petersen *et al*., 2023; Decaestecker *et al*., 2024).

Overall, evaluating the responses of externally activated molecular pathways within individuals and communities will allow for understanding the diverse effects of climate change on the expression of phenotypic plasticity at multiple levels of biological organisation. Recently, such large‐scale approaches have become more feasible due to the low cost of high‐throughput gene expression analysis, which can be used to study the genomic reaction norms in common‐garden experiments and allows for integrating multiple species and biotic and abiotic conditions to elucidate the molecular mechanisms of phenotypic plasticity (e.g. Oomen & Hutchings, 2022).

### (2) The importance and constraints of different aspects of phenotypic plasticity in a changing climate

Activation of the molecular pathways of plastic traits can result in different forms of phenotypic plasticity which vary in the type of traits affected, in the emergence, duration and permanence of the plastic traits (irreversible *versus* reversible), as well as in their response type (anticipatory *versus* responsive, and active *versus* passive) (Whitman & Agrawal, 2009; Figs 1 and 2, bottom row). These differences affect the relative importance of the different aspects of phenotypic plasticity as a buffer against climate change on the community level, which is discussed in Section III.

#### (a) Will anticipatory and irreversible phenotypic plasticity decline under climate change?

Anticipatory phenotypic plasticity requires reliable cues for organisms to predict future climatic conditions accurately and to adjust the phenotype ahead of the environmental change (Whitman & Agrawal, 2009; Bernhardt *et al*., 2020). However, climate change and associated extreme climatic events (e.g. heat waves, droughts) lead to more disrupted and unreliable environmental cues (reviewed by Bonamour *et al*., 2019). Whether anticipatory phenotypic plasticity will become unreliable under climate change or will still lead to adaptive phenotypes depends on the timescale anticipated by the individual (e.g. circadian, seasonal, annual, decadal), the temporal and spatial autocorrelation of the available environmental cues and the plastic phenotype (Scheiner, 2013), the way the cues change (e.g. gradually, abruptly, change of information that the cue provides), the cue's distinctiveness (i.e. unique, redundant; Bonamour *et al*., 2019), as well as the timing and duration of the plastic trait (rate of change; labile *versus* irreversible traits).

**Fig. 3:**
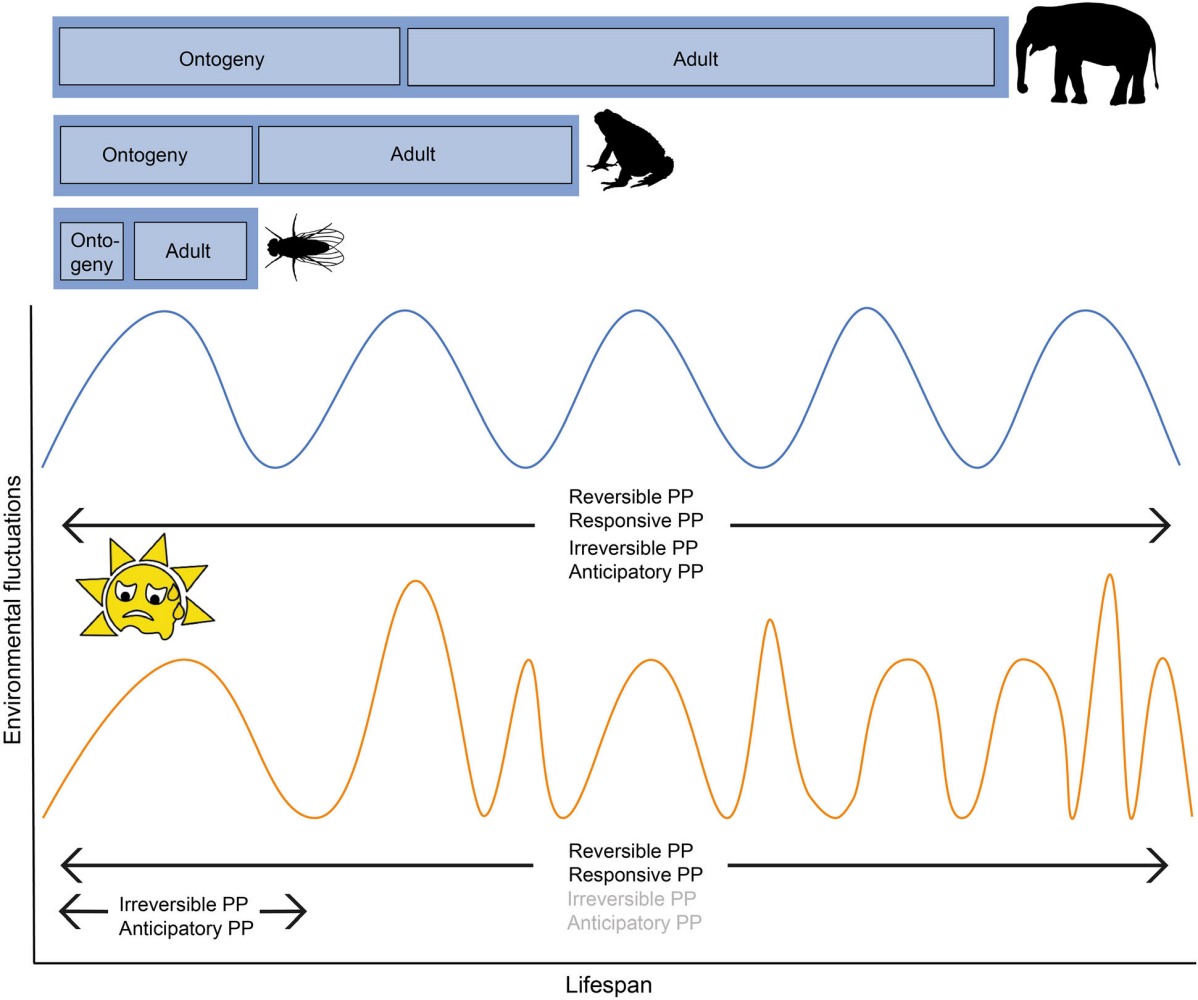
Proposed shift of phenotypic plasticity types relative to length of ontogeny and lifespan under climate change. Without climate change, the environmental fluctuations and associated cues are more predictable (illustrated by the blue, more regular waves) and all types of phenotypic plasticity (PP) may be used across the whole lifespan of long‐ and short‐lived organisms (arrows). Under climate change, the environmental fluctuations (in particular weather extremes) and associated cues (illustrated by orange waves with more irregular amplitude and frequency) become less predictable. The same environmental fluctuation can be perceived differently depending on the organism and the spatio‐temporal scale (i.e. resolution) at which it interacts with the environment. Larger organisms generally have longer lifespans, influencing the perceived magnitude of change. When faced with the same environmental fluctuation, smaller short‐lived animals with faster metabolism may perceive it as a major cue, whereas larger long‐lived animals with slower metabolic rates may perceive it as environmental noise or as an oscillating stressor. Therefore, a potential stressor only acquires meaning depending on the focal timescales at which the organism operates and its generation time (e.g. Einum & Burton, 2023; Dupont *et al*., 2024). Thus, in long‐lived organisms such as African elephants that live up to 70 years, have a 2‐year gestation period and mature after *ca*. 15 years, reliance on irreversible PP both during ontogeny and adult life could lead to maladaptation and irreversible PP traits may therefore decrease under climate change (grey text). In toads that live about 10–12 years, the tadpole development of a few weeks is more likely to fall into a predictable time window, where the anticipated environment at egg laying matches the environment during tadpole development, while the long‐lived adult will more likely face unpredictable environmental fluctuations. In this case, different types of PP will be relevant for different life stages (arrows). Anticipatory PP can remain adaptive in long‐lived animals, for example if the cue triggers a reversible response and the duration of the plastic phenotype falls into a time window with environmental conditions matching the cue (typically a short‐term, rapid behavioural or physiological response following the cue), or an induced irreversible trait is still adaptive or neutral in the novel condition. However, we predict an overall decline in anticipatory PP (grey text). Short‐lived organisms such as *Drosophila* flies that can complete their life cycle during a few weeks within predictable environmental conditions can usually rely on all forms of PP (arrows), including irreversible and anticipatory PP. Ultimately, the repertoire of different plastic responses used by a species will depend on the costs and benefits of each plasticity type for the organism and the context of local climate change drivers. The lifespan and stages of the different organisms are not to scale.

We suggest that taxa with short ontogeny and a short lifespan relative to the timescale of environmental fluctuations (such as heat waves, drought or seasonal predators) can rely on anticipatory phenotypic plasticity, since the current and developmentally or maternally anticipated environments most likely match the actual, encountered conditions over the whole lifespan of the organism (Fig. 3). This is supported by studies on the predatory mite *Phytoseiulus persimilis* (Le Hesran *et al*., 2020). After 24 h, females exposed to dry conditions start producing drought‐resistant eggs that hatch after 7–14 days (Vangansbeke *et al*., 2013). The offspring are thus adapted to a new environment at a very short timescale. Long‐lived species can also rely on anticipatory phenotypic plasticity if they have a short ontogenetic stage and a short time lag between cue perception and phenotype expression, leading to strong temporal autocorrelation. For example, tadpoles of the long‐lived western spadefoot toad *Pelobates cultripes* accelerate their development by 30% in anticipation of desiccation in response to a decreasing water level in the pond (Gomez‐Mestre, Kulkarni & Buchholz, 2013).

Conversely, the role of anticipatory phenotypic plasticity leading to irreversible trait change should decrease in species with long lifespans, because individuals can become maladapted throughout much of their lifespan if they later encounter a different environment (e.g. Degut *et al*., 2022; Fig. 3). Maladaptation may be mitigated by learning and behavioural changes as shown in the well‐studied example of anticipatory phenotypic plasticity in the great tit, which adjusts its breeding time to the life cycle of the winter moth *Operophtera brumata* to feed its chicks (Charmantier *et al*., 2008). A recent predictive model suggests, however, that the tit's plastic behaviour (earlier egg laying) will be insufficient and lead to a population decline if the phenological asynchrony between the tit's breeding time and the moth's hatching increases by more than 24 days (Simmonds *et al*., 2020). This may render the current anticipatory plasticity affecting egg‐laying behaviour maladaptive unless it can evolve to reduce the phenological asynchrony.

On the other hand, new forms of adaptive plasticity can evolve or changes in other plastic traits may allow responses to novel environmental conditions (orange symbols in Fig. 1). The latter mechanism can be illustrated by a (hypothetical) example, in which warming‐induced reduction in body size of an aquatic predator allows it to feed on smaller prey that was previously either not profitable or difficult to catch. This would allow the predator to replace the prey lost due to for example phenological asynchrony or, more generally, offset the increased metabolic demands at higher temperature. Additionally, the predator's current anticipatory plasticity – affecting the predator's phenology or migration patterns – could be adaptive for exploiting this novel prey.

Both anticipatory and responsive phenotypic plasticity can be further impacted by a disruption of cue perception due to the direct or indirect consequences of climate change (reviewed by Bonamour *et al*., 2019). Circadian rhythms for instance typically function as a form of anticipatory plasticity. Recent investigation in aquatic organisms suggests significant interactions between disturbed circadian rhythms and climate‐change‐related stressors, such as hypoxia. In zebrafish *Danio rerio*, for example, hypoxia‐inducible factor 1 binds to the promoter region of the circadian clock gene *Per* and thus competes with the binding sites of transcriptional regulators of the circadian clock, leading to a decrease of *Per* transcription in hypoxic conditions. This can lead to an impaired circadian rhythm, even if the right cues (e.g. light) for maintaining the circadian cycle are present (Prokkola & Nikinmaa, 2018). An example of cue disruption in responsive phenotypic plasticity is the exposure of the cleaner fish *Ctenolabrus rupestris* to future levels of CO_2_ (Sundin & Jutfelt, 2016). *C. rupestris* becomes indifferent to predator odour and displays reduced responsive predator avoidance plasticity in the high‐CO_2_ environment.

During ontogenesis, both anticipatory and responsive plasticity are further constrained by the timing of the environmental cue, which must overlap with a sensitive period to elicit a switch to alternative developmental pathways (e.g. Sieriebriennikov *et al*., 2018). Furthermore, the sensory modalities for cue detection must be present in the corresponding life stages for plastic phenotypes to form (Bernhardt *et al*., 2020). Thus, changes in the length and timing of development due to climate change might interfere with the ability to perceive the environmental cue. A systematic analysis of the relationship between developmental plasticity, generation time, life stages, lifespan and sensitivity periods across the domains of life is needed to understand fully the importance of anticipatory phenotypic plasticity in the context of climate change. Advances in understanding these relationships have been made in the study of the anticipatory formation of defence structures in the freshwater crustacean *Daphnia*. For example, in *D. longicephala*, Weiss, Leimann & Tollrian (2015) have determined the pathway to cue perception, the sensitivity windows to predator kairomones, the developmental timeframes and the time lag between cue perception and the induced defence structure. This knowledge paves the way for predicting under which future conditions the pathway to anticipatory plasticity might be disturbed or might still result in adaptive defence structures.

Furthermore, an irreversible plastic response in one trait may constrain the possibility to express other plastic traits. For example, warming may both increase growth rates in ectotherms and lead to earlier maturation at smaller size, which could also lead to irreversible plastic changes in the relative proportions of organ sizes and the timing of developmental events such as ossification. The resulting changes in body size and shape can constrain the range of other trait types such as behaviour and potentially make other ‘non‐target’ traits irreversible.

The previous examples suggest that the role of anticipatory plastic phenotypes might decline under future climate change, potentially leading to an overall decrease of plastic phenotypes or an increased reliance on (reversible) responsive phenotypic plasticity (see Fig. 1 for a hypothetical example). The disadvantage of responsive phenotypic plasticity is that damage (leading to lower fitness) might occur before the phenotype can be changed (Whitman & Agrawal, 2009). For example, plants responding to the saliva of herbivores by producing toxins will have damaged leaves before they can fend off the attack (reviewed by Halkier & Gershenzon, 2006; Fig. 1). Consequently, if anticipatory plasticity becomes maladaptive or unavailable for certain traits, the increasing use of responsive phenotypic plasticity under climate change could reduce fitness.

#### (b) Effects of warming‐induced passive and active phenotypic plasticity on life histories and population structure and growth

Both passive and active phenotypic plasticity play an important role in individual‐level responses to climate change (Figs 1 and 2). For example, warming typically leads to faster individual growth and smaller maturation size in many ectotherms [temperature–size rule; (Atkinson, 1994; Tan *et al*., 2021; Huss *et al*., 2019)]. Several studies reported that warming can directly influence the physiological decision in ectotherms to mature (Tobin & Wright, 2011), shifting maturation towards smaller sizes (Grift *et al*., 2003). Interestingly, warming may also change life‐history traits (e.g. body size, developmental time) indirectly *via* the microbiome (Stock *et al*., 2021; Kikuchi *et al*., 2016), although this mechanism is much less understood and studied. Warming also indirectly affects fecundity by altering the energy budgets of individuals (i.e. by increasing metabolic costs) and by affecting body size (e.g. Fryxell *et al*., 2020; Clark *et al*., 2021). Although larger individuals have higher fecundity, smaller individuals are more likely to have larger relative investment in fecundity (Wootton *et al*., 2022) as predicted by life‐history theory (Stearns, 2000).

These warming‐induced plastic effects on individual life histories can alter population structure. Smaller individuals should be relatively more abundant (i.e. population size spectrum should have a steeper slope) in warmer environments if the maximum body size decreases with warming, although this can be countered by other indirect effects of warming on size‐dependent individual growth and mortality rates (Lindmark, Karlsson & Gårdmark, 2023; Dijoux *et al*., 2024a). Interestingly, warming can shift the population structure towards the dominance of adults over juveniles when both stages compete for shared resources, even if smaller species are favoured over larger ones in warmer environments (Uszko, Huss & Gårdmark, 2022). Phenotypic plasticity should also lead to higher intrinsic population growth rates if the positive effect of earlier maturation and thus shorter generation times outweighs the negative effect of smaller maturation size and hence lower absolute fecundity or higher mortality (Lindmark *et al*., 2023). However, quantitative analyses of these effects and their correlations are required to predict the overall effect of warming‐induced plastic responses on the fitness of different taxa. This can be done by using integral projection models linking an individual's state to its growth, survival or reproduction (Merow *et al*., 2014; Vindenes *et al*., 2014; Fung *et al*., 2022) and physiologically structured population models based on dynamic energy budgets to link organismal processes to population dynamics (e.g. Chaparro‐Pedraza & de Roos, 2021; Dijoux *et al*., 2024b).

## III. EFFECTS OF CLIMATE CHANGE ON SPECIES INTERACTIONS AND COMMUNITIES

Effects of global warming on individuals ultimately translate into changes in species interactions and community structure and dynamics (Fig. 2). Plastic responses to warming such as reduction in body size (Daufresne, Lengfellner & Sommer, 2009; Sentis, Binzer & Boukal, 2017; Tan *et al*., 2021), phenological matches/mismatches (Durant *et al*., 2007; Dakos *et al*., 2019) and distributional range shifts can reshape the strength and type of species interactions and ultimately threaten population persistence (Pinsky, Selden & Kitchel, 2020), particularly for consumers at higher trophic levels (reviewed by Boukal *et al*., 2019). Studying phenotypic plasticity in the context of species interactions is challenging given the great diversity of traits that organisms induce in response to multiple axes of environmental variation, such as the simultaneous effects of warming and increased predation risk. In the following, we outline how the effects of climate warming on phenotypically plastic traits affect species interactions and discuss the role of individual‐level variation and phenotypic plasticity in community‐level responses to warming. We will also highlight the consequences of those responses for regime shifts and the predictability of the effects of warming on community structure and dynamics.

### (1) Consequences of warming‐induced passive phenotypic plasticity

Variation of metabolic rate with temperature can explain the thermal sensitivity of other processes on individual, population and ecosystem levels (Brown *et al*., 2004). Previous work building on passive metabolic plasticity showed that respiration is more sensitive to warming than production (Allen, Gillooly & Brown, 2005; López‐Urrutia *et al*., 2006; Yvon‐Durocher *et al*., 2010). A recent synthesis of a new theoretical framework and empirical evidence indicated that differential temperature sensitivities of biological rates among different taxonomic groups lead to stronger top‐down control in aquatic ecosystems (Bideault *et al*., 2021; O'Connor *et al*., 2009; Kratina *et al*., 2012), a higher proportion of aquatic heterotrophs relative to autotrophic biomass (Yvon‐Durocher *et al*., 2011; Shurin *et al*., 2012), and more top‐heavy terrestrial food webs in warmer environments (De Sassi & Tylianakis, 2012). On the other hand, top predators often suffer from starvation when their metabolic demands increase faster than their ingestion rate with warming (Rall *et al*., 2010). This can lead to gradual declines in top‐predator populations with warming, but also to an abrupt collapse above a certain temperature threshold due to an emergent Allee effect (Lindmark *et al*., 2019). Interestingly, the plastic reduction in body size of top predators induced by temperature can buffer this starvation effect by increasing ingestion relative to metabolic losses (Sentis *et al*., 2017).

### (2) Role of active phenotypic plasticity and individual‐level variation in consumptive interactions under warming

Climate warming can interfere with phenotypically plastic responses of organisms to their predators and to predation risk. For example, many species mature at smaller body size under warming (Atkinson, 1994). Smaller body size is also actively induced in response to increased predation mortality (Stoks *et al*., 2016), while larger body size is actively induced in many aquatic invertebrates to evade gape‐limited predators (Kratina, Hammill & Anholt, 2010; Beckerman, Rodgers & Dennis, 2010). Predation risk and warming can thus have consistent or contradictory effects on body size. Climate warming may also disrupt active plastic behavioural responses such as predator‐induced dispersal and anti‐predator behaviour (see Section II.1) in ways that either strengthen or reduce predation pressure on prey populations.

Consumers with more plastic behaviour may be less affected by changing species interactions and ultimately benefit from warming. Active plasticity of consumers in resource use can lead to prey switching (Murdoch, 1969; Greenwood & Elton, 1979; Elliott, 2004), which can stabilise communities and promote coexistence in simple predator–prey models (Oaten & Murdoch, 1975; Chesson, 1984) and in complex food web models (Pelletier, 2000; Kondoh, 2003). Climate change can also reduce prey availability through phenological mismatches or spatial decoupling through asymmetric range shifts. Generalist predators with flexible prey use can cope better with such apparent declines in prey than specialist predators with low trophic plasticity (e.g. Damien & Tougeron, 2019; Barrientos, Bueno‐Enciso & Sanz, 2016; Neves *et al*., 2021).

This “rescue” effect of active plasticity in resource use probably also applies to other types of biotic interactions such as pollination, herbivory and parasitism, where the fate of associated species depends strongly on the dynamics of their host species (Koh *et al*., 2004). However, the intimately co‐evolved relationships between mutualists, between parasitoids and their hosts, and between microbiomes and their hosts are often more specialised than those between predators and their prey, leading to lower connectance in parasitoid–host networks as compared to predator–prey networks (Van Veen *et al*., 2008). This suggests that ecological networks dominated by parasitoid and mutualistic interactions may be more sensitive to climate warming than food webs dominated by more generalist consumers with a broader range of potential resources and flexible resource use.

The effects of global warming on species interactions can be further exacerbated or mitigated by individual‐level variation in phenotypically plastic traits driven by micro‐environmental or genetic differences. This intraspecific variation may be substantial and dominate the predicted effects of warming on species interactions, population stability and evolutionary potential. For example, the behavioural component of trophic link strength in a dragonfly–newt larvae system can vary much more among individuals within the same treatment (temperature, presence/absence of predation risk cues) than between treatments (Gvozdik & Boukal, 2021). Between 10 °C and 30 °C, the behavioural component was the main driver of variation in the stability of predator–prey dynamics parameterised with the experimental data in that dragonfly–newt larvae system. On the other hand, warming typically reduces the metabolic scope of individuals, defined as the difference between the maximum and standard metabolic rates (Sandblom *et al*., 2016), and hence should reduce the individual‐level variation in phenotypic responses to stressors such as predation risk if these responses rely on surplus capacity (Pink, Abrahams & Krkošek, 2016; Šupina, Bojková & Boukal, 2020). In other words, a reduced metabolic scope leaves less energy to invest in active plastic responses, which should reduce the phenotypic variation among more or less plastic genotypes. How these canalised responses alter the strength of trophic interactions and food web dynamics will depend largely on their impacts on consumer and resource behaviour, phenology and life histories.

### (3) Importance of active and passive phenotypic plasticity for competitive interactions under warming

Although most research has focused on the role of phenotypic plasticity in consumptive interactions, recent studies show how plasticity can alter competitive interactions and species coexistence. Some studies found that phenotypic plasticity can promote species coexistence by reducing the negative influence of competition *via* greater niche differentiation (Hess *et al*., 2022; Hendry, 2016; Turcotte & Levine, 2016). Conversely, it has also been argued that plasticity helps dominant species to maintain their competitive advantage across multiple environments (Pérez‐Ramos *et al*., 2019). Modern coexistence theory resolves this contradiction by suggesting that plasticity promotes stable coexistence when it strengthens niche differences more than differences in average fitness (Turcotte & Levine, 2016). Some traits, however, may influence both niche and fitness differences among species (Kraft, Godoy & Levine, 2015). As a result, trait plasticity in response to competition can either promote or hinder coexistence in competitive communities.

Active and passive phenotypic plasticity under future climate warming may lead to more asymmetric competition and communities characterised by the dominance of a few strong competitors or to greater opportunities for niche differentiation and coexistence among competitors. A study of competing plant communities found that rapid trait changes in response to a shift in the competitive environment can promote coexistence in ways not captured by the usual measures of niche differentiation (Hess *et al*., 2022). Another recent field study demonstrated that plasticity in light‐ and water‐use traits enabled plant species to maintain their position in the competitive hierarchy across warming (and drought) climate conditions (Pérez‐Ramos *et al*., 2019). Plasticity in two physiological traits related to light and nitrogen acquisition acted to increase competitive inequalities among species, destabilising their coexistence. At the same time, plasticity in light‐spectrum‐use and phenology‐related traits generated niche differences among species, stabilising their coexistence (Pérez‐Ramos *et al*., 2019). Future studies should elucidate whether active and passive phenotypic plasticity in response to climate warming is greater for traits related to competitive inequalities or niche differentiation among species to predict better the impacts on competitive interactions under warming.

### (4) Role of active and passive phenotypic plasticity in community structure and dynamics

Since complex natural communities encompass a variety of life forms, we need to extend the study of active and passive phenotypic plasticity to cover a broad diversity of taxonomic groups and species interactions and evaluate the role of phenotypic plasticity in multitrophic networks with different interaction types (e.g. Pilosof *et al*., 2017). This includes the need to consider intraspecific variation in ecological interactions and physiological processes – both within individuals during ontogeny and between individuals, for example due to genetic differences – as community dynamics emerge from interactions among individuals (e.g. Gårdmark & Huss, 2020). The integration of the role of phenotypic plasticity at multiple trophic levels, ranging from individuals to communities, would increase the realism of future studies, but also the complexity of theoretical frameworks (Fig. 2). For instance, the coexistence of multiple competitors depends on niche or fitness differences (see Section III.3) as well as on indirect feedbacks across multiple trophic levels that all can alter competition strength, for example in apparent competition.

To address this complexity, we propose the combination of two complementary approaches (Fig. 2): (*i*) a bottom‐up approach that reduces complexity at the community level but tries to embrace the relevant processes at the individual or population level and can study mechanisms and interactions in full detail; and (*ii*) a top‐down approach that embraces the full complexity of higher ecological levels (e.g. community composition or food web structure) and explores their changes over time or ecological gradients, while being less able to study the different processes and how they interact in detail. Both top‐down and bottom‐up approaches can bring important insights on the role of phenotypic plasticity on individual, population and community responses to climate change. We argue that efforts to integrate these two approaches systematically would provide a more comprehensive understanding of the role and impact of phenotypic plasticity at the whole‐community and ecosystem level.

#### (a) Bottom‐up approach

Bottom‐up approaches start from processes at the individual and population level, considering cellular or trait trade‐offs of plasticity, and quantifying how such trade‐offs change population dynamics. It often starts with a simple ecological system, generally limited to one species or to simple community modules consisting of two interacting species, where mechanisms and their consequences can be easily identified. This understanding can be extended by adding more species or environmental drivers and considering more traits. From an experimental perspective, techniques such as artificial selection or genetic engineering could be used to manipulate levels of phenotypic plasticity of a given species and quantify its consequences for population and community dynamics (Forsman, 2015; Krebs, 1998; Ketterson *et al*., 1996). However, this approach is currently only feasible for a limited number of model organisms for which the genes and pathways of the plastic response are known. From a modelling perspective, the bottom‐up approaches are tractable and highly versatile, and can accommodate any assumptions on whether the plastic responses are passive or active, adaptive or non‐adaptive, and what trade‐offs exist between traits. Moreover, assumptions on whether plasticity is regulated by a single cue or by a complex integration of multiple cues, and how sensitive phenotype expression is to these cues, can be explicitly incorporated.

**Fig. 4:**
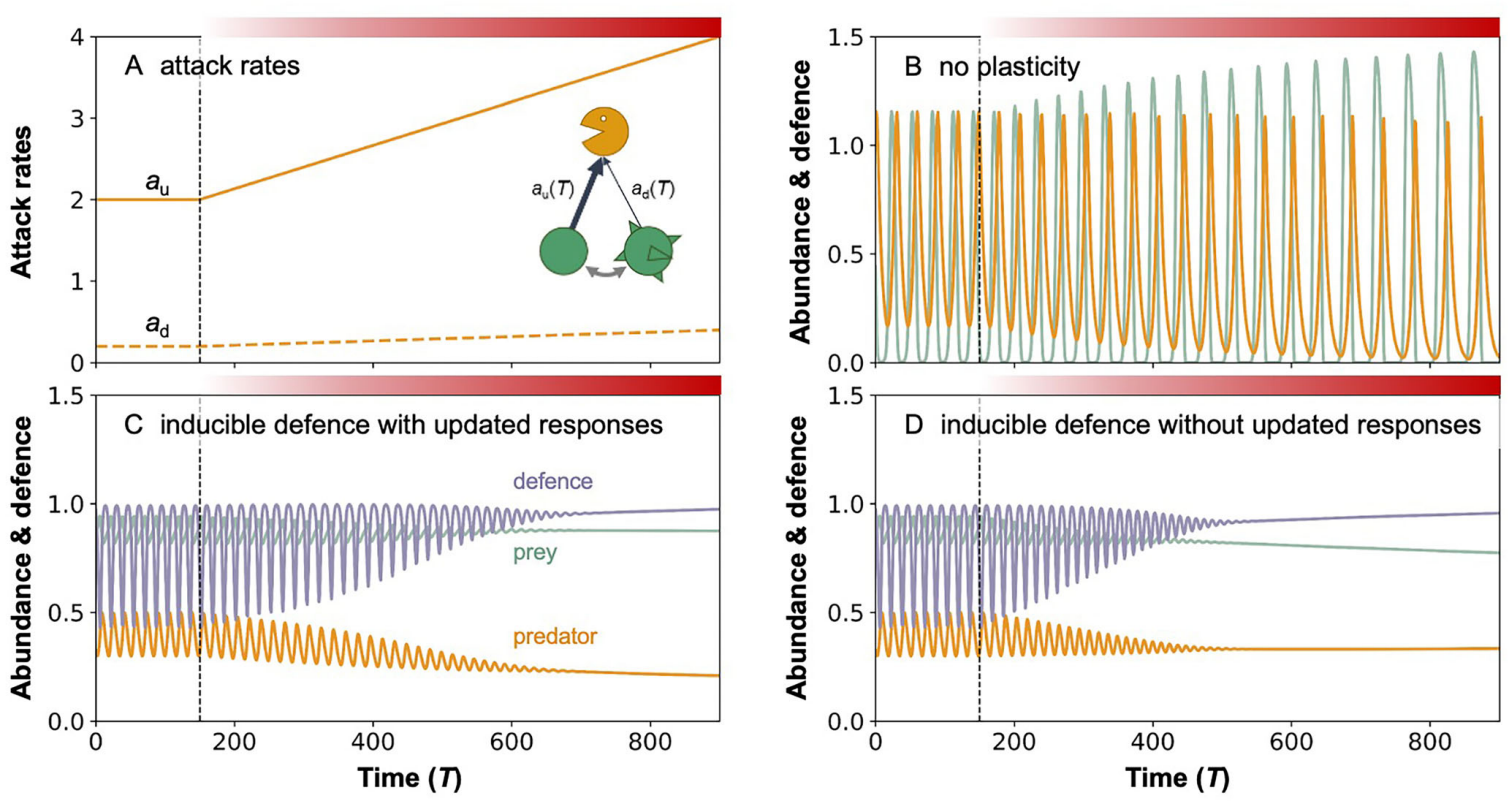
Dynamics of a predator–prey model with an active inducible antipredator response (see Yamamichi *et al*., 2019) that we extended to include temperature dependence in the attack rates. The dashed vertical line indicates a distinction between the dynamics before (time < 150) and during (time > 150) environmental change; the colour intensity of the red bar above each panel indicates the severity of warming. (A) Attack rates of the predator on undefended (*a*_u_) and defended (*a*_d_) prey phenotypes. With gradual warming (between times 150 and 900), both attack rates gradually increase twofold. (B) Impact of warming on the dynamics without plasticity: increased overexploitation by the predator results in more severe cycles. (C, D) Impact of reversible plasticity on the dynamics, showing a stabilising effect that is counterintuitively enhanced by the effect of warming. In both C and D, prey estimate predation risk by the abundance of predators and their conspecifics (Tollrian *et al*., 2015) to determine whether they should express a defended or undefended phenotype. In C, the model also incorporates the increased attack rate (i.e. individuals consider the same predator density at higher temperature to constitute a higher predation risk), while in D, their choices are based only on the predator and prey abundances.

As an illustrative example, Fig. 4 shows a model representing a simple community consisting of one prey and one predator species, where the prey has an active plastic antipredator defence. Prey expresses an undefended or a defended phenotype based on the perceived predator and conspecific densities, which together form a proxy for predation risk (Tollrian *et al*., 2015). Models of this type have been extensively used to show, for example, how inducible defences stabilise predator–prey dynamics (compare Fig. 4B–D, time 0–150; Vos *et al*., 2004; Cortez, 2011; Yamamichi *et al*., 2019; Yamamichi, Yoshida & Sasaki, 2011). As an illustration we extend this model to include effects of global warming (Fig. 4, time > 150) by incorporating thermal dependence of model parameters (Sentis *et al*., 2017; Vasseur & McCann, 2005). For this simple demonstration, we first assume that warming may cause a passive increase in predator activity, increasing the attack rate on the prey (Fig. 4A), while no other parameters are affected. Without active plasticity in the antipredatory response of the prey, this has a destabilising effect on the dynamics (Fig. 4B), giving the intuitive expectation that warming will reduce the stabilisation resulting from plasticity. However, this model can be used to show that the reverse may also occur (Fig. 4C, D). It may also be used to show the impact of reduced cue reliability, by comparing the dynamics generated by a prey that recognises the increased predation risk that each more active predator represents (Fig. 4C) with a prey that continues to use the same abundance‐based cues (Fig. 4D). In the latter case, the prey uses a less precise estimator for predation risk, resulting in a slightly lower prey abundance and a strongly increased predator abundance. Such models are thus highly useful in challenging our intuitions on the general effects of warming on community dynamics and allow the exploration and direct comparison of a wide variety of potential scenarios.

Many further model extensions are possible: including additional environmental gradients, increasing vertical or horizontal diversity with for example a second predator (against which the prey may have a second induced defence, which may be incompatible with the first). Moreover, the molecular mechanisms leading to the expression of the induced phenotype can be represented in the model by linking molecular pathways to model parameters. With detailed knowledge of a particular system, especially on the regulation of the plastic response and how it changes with increased temperature, such models may also be tailored to predict responses of simple communities to global warming. However, the extension to increasingly complex communities makes it mathematically less tractable and it may become very difficult to trace down the mechanisms driving the observed patterns. Agent‐based models may allow inclusion of more dimensions of complexity, but at the cost of a less‐complete exploration of how the different mechanisms precisely interact.

#### (b) Top‐down approach

Within a community, both intra‐ and interspecific processes, and their interactions simultaneously shape community dynamics. Phenotypic plasticity can alter the relative importance of intra‐ and interspecific trait variability along a thermal gradient (Jacob & Legrand, 2021), which further complicates our understanding of how plasticity impacts community structure and dynamics as discussed in Section III.1–3. A top‐down approach embraces the full complexity of the community and aims to quantify the relative importance of different mechanisms in determining dynamics and patterns.

One approach is to consider the trait variation observed at the community level and disentangle this trait variation into components of phenotypic plasticity, genetic and species variation. Partitioning methods have been developed to quantify the contribution of plasticity, evolution and species abundance change to (*i*) shifts in community mean trait values over time based on the Price equation or on reaction norms (Govaert, Pantel & De Meester, 2016), or to (*ii*) the community trait variation along an environmental gradient of interest (Brans *et al*., 2017; Lajoie & Vellend, 2018). When the goal is to quantify the contribution of phenotypic plasticity, the assessment of reaction norms obtained from common garden or transplant experiments is needed. Reaction norms have formally been used to provide a link between the genotype and the environment (Woltereck, 1909) but can also be quantified at the population level (Stoks *et al*., 2016). However, conducting common garden or transplant experiments for all species within a community or set of communities may often become time‐consuming or infeasible.

While partitioning approaches allow for a relatively straightforward evaluation of the relative importance of different processes operating at different levels of ecological organisation, they reveal little information on the causes of these contributions (except when combined with targeted laboratory experiments). Importantly, the results are sometimes counterintuitive, because the importance of phenotypic plasticity of a particular species for community dynamics not only depends on the amplitude of its phenotypic plasticity but also on its relative abundance and its interaction strengths with other species in the community. This is where a top‐down approach can make a difference: while a bottom‐up approach provides proof of principle of how the phenotypic plasticity of a given species might influence responses in nature, a top‐down approach, although limited in the detail, can give indications as to how important phenotypic plasticity is under natural conditions.

However, differentiating between phenotypic plasticity and other processes (e.g. microevolution) as well as assessing the drivers of phenotypic plasticity requires targeted experimental work, such as carefully designed common garden (e.g. Brans *et al*., 2017) or transplant (e.g. Lajoie & Vellend, 2018) experiments. For example, common garden experiments could be designed to quantify the phenotypic plasticity induced by specific environmental conditions by placing the focal species within different common gardens, or to quantify the contribution of phenotypic plasticity to the community composition by placing the focal species in a common garden within the larger community. However, such an approach often only allows for a quantification of the trait variation and a bottom‐up approach might be needed to deduce the pathways leading to the phenotypic plasticity observed. Alternatively, low‐ and high‐plasticity lineages can be created using genetic engineering, RNA interference methods or artificial selection and experimental evolution (Turcotte & Levine, 2016; Schaum *et al*., 2022). These lineages can then be used in experiments to disentangle the contributions of plasticity to observed community responses to climate change. However, while artificial selection is feasible in species with short generation times (e.g. phytoplankton; Schaum *et al*., 2022) and known genetic variation for plasticity [e.g. aphids (Deem *et al*., 2024; Sentis *et al*., 2019)], the manipulation of gene function at a multi‐species level would require a considerable increase in our knowledge of the genes regulating plasticity.

An overlooked factor in studying changes in community‐ and ecosystem‐level dynamics is the host microbiome. Given its effect on the host phenotype, and especially for organisms that are key for structuring ecosystem dynamics, microbiomes can indirectly affect host community dynamics in a bottom‐up and top‐down approach. The gut microbiome shapes ecological–evolutionary (eco‐evo) dynamics in the host community through its effects on the host phenotype (Decaestecker *et al*., 2024). Complex eco‐evo feedback loops between the gut microbiome and the host communities might thus be common. Bottom‐up dynamics occur when eco‐evo interactions shaping the gut microbiome affect host phenotypes with consequences at population, community, and ecosystem levels. Top‐down dynamics occur when eco‐evo dynamics shaping the host community structure the gut microbiome (Decaestecker *et al*., 2024). We thus argue that, in general, the integration of both approaches will allow incorporating the complexity of natural systems (i.e. multiple species, traits and environmental factors), while being able to identify the mechanisms driving the observed patterns and to assess the relative contribution of phenotypic plasticity to responses to climate change.

This integration can be done using controlled laboratory experiments or theoretical modelling. For example, one can develop experiments including several species, genotypes and environmental drivers where the level of plasticity of the different species can be manipulated. This could be done by knocking down genes encoding the plastic trait (when the trait is mostly monogenic) or by manipulating the genetic composition of the population towards more or less plastic genotypes. Choosing such genotypes might not always be straightforward when considering multiple traits. However, one could investigate the multivariate trait space and choose genotypes with smaller or larger total trait space. Theoretical models can also be used to assess the role of plasticity for species and community responses to climate change in complex communities. We propose to use a bottom‐up approach based on agent‐based models (ABMs), that can readily incorporate individual‐level processes (Railsback & Grimm, 2019). The predictions of these ABMs could then be compared to the contributions calculated from partition methods often used in a top‐down approach. Combining both bottom‐up and top‐down approaches could inform about the underlying mechanisms driving the observed contributions obtained by the partition methods.

### (5) Predicting community responses and regime shifts

The predictability of community responses to climate change will depend on the dominant type of plasticity. We expect that passive plasticity, resulting from fundamental physical and chemical constraints, will lead to more predictable responses than active plasticity, where organisms have evolved a variety of plastic responses triggered by environmental cues. For instance, the rates of chemical reactions are strongly temperature dependent, so that the metabolic rate of most ectotherms increases with a similar slope to warming (i.e. a passive plastic response; Brown *et al*., 2004). The effects of passive phenotypic plasticity could thus be readily incorporated into models of community dynamics to enable predictions of ecological dynamics (Petchey *et al*., 2015; Pennekamp *et al*., 2019), and many such models already exist (e.g. Sentis *et al*., 2017; Uszko *et al*., 2022; Lindmark *et al*., 2019; Binzer *et al*., 2016; Osmond *et al*., 2017; Dijoux *et al*., 2024b).

Active plastic responses to the same environmental cue can induce very different phenotypic responses in different taxa (see Section II.1). This makes community dynamics less predictable, as different individual‐level phenotypic responses can translate into different community‐level interactions. Active plasticity should then shorten the ecological forecast horizon that can be achieved for such communities (Petchey *et al*., 2015). This also means that existing insights on the impacts of climate change on communities, obtained from models that do not include some important aspects of active plastic responses, may be biased (Sentis, Morisson & Boukal, 2015). A recent conceptual synthesis further predicts an increased importance of stochastic processes in community assembly in response to environmental warming (Saito, Perkins & Kratina, 2021). The theory indicates that reduced longevity and population sizes, together with the increased intrinsic mortality and the community biomass turnover in warmer environments, should make community assembly of ectotherms more stochastic and less predictable than in colder environments (Saito *et al*., 2021), and our limited understanding of the community‐level consequences of active plastic responses to warming may amplify this uncertainty.

**Fig. 5:**
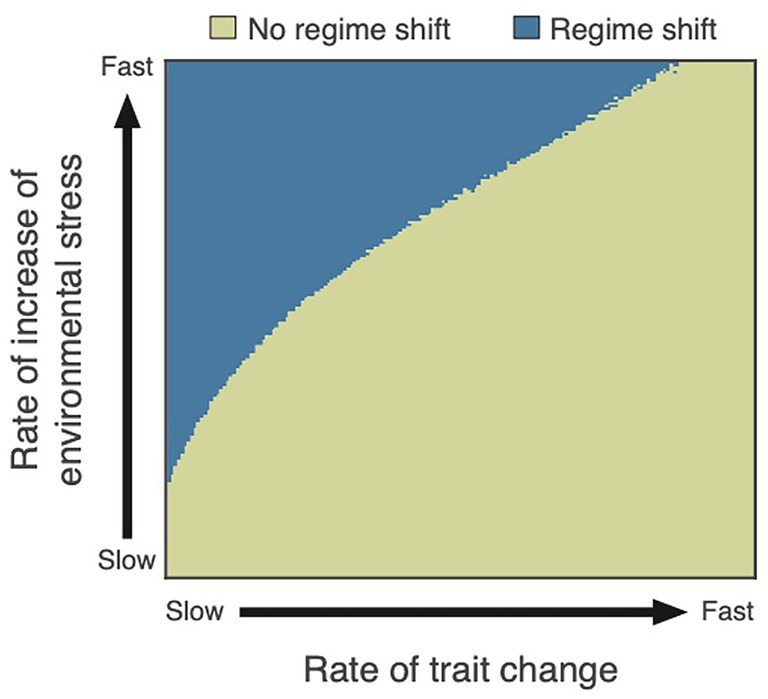
Regime shift as a function of rates of trait change and of environmental stress increase. Although the risk of regime shifts increases with faster environmental change, this risk can be reduced by rapid phenotypic trait changes. Phenotypic trait changes can shift a critical threshold to higher levels of environmental stress, which increases stress tolerance. Therefore, the speed of trait change determines the rate at which the system (e.g. population, community, ecosystem) becomes more tolerant to stress, avoiding tipping into an alternative contrasting state (from Chaparro‐Pedraza, 2021).

Research on phenotypic plasticity at the community level should also consider the potential for climate change to trigger regime shifts that can have significant consequences at the ecosystem level. Biological systems do not always respond to gradual environmental change in a smooth manner, but abrupt, catastrophic transitions may occur in response to gradual changes when a critical threshold is exceeded (Scheffer, 2001). Critical thresholds and regime shifts between alternative states of the system depend on individual‐level sensitivity to environmental stress (Mori, Furukawa & Sasaki, 2013), which is mediated by phenotypic traits (Vellend & Geber, 2005). Selective pressures altered by environmental change can drive rapid changes in phenotypic traits underlying the sensitivity to environmental stress. Recent work examining the effects of evolutionary trait changes on the risk of regime shifts found that rapid trait changes can significantly reduce the risk of ecosystem regime shifts (Chaparro‐Pedraza, 2021, 2024; Fig. 5). This implies that phenotypic plasticity could reduce the risk of ecosystem regime shifts by driving trait changes faster than evolution. Warming can therefore either increase or decrease the propensity for ecological regime shifts, depending on which traits are plastic and whether their plasticity is enhanced or reduced by warming, see Section III.4.*a* for examples of prey responses to predation risk and *Dijoux et al*. (2024b) for a recent simulation study.

## IV. OUTSTANDING QUESTIONS

Table 2 highlights multiple under‐studied topics related to the role of phenotypic plasticity in individual, population and community responses to warming.

**Table 2:** Outstanding questions for future research. The grey bars indicate the (overlapping) biological levels corresponding to the open questions.

Level			Open questions
Individual			Which (combination of) environmental cues are required to produce plastic traits in specific contexts and how does climate change influence the availability, reliability and persistence of these environmental cues?
			How are the changed/novel environmental signals perceived and transmitted from upstream cue‐sensing regulators to the appropriate cells, tissues and organs, that produce the alternative phenotypes, and which downstream regulators are involved?
			In what ways will the different types of phenotypic plasticity and the underlying molecular mechanisms constrain the production of adaptive phenotypes under climate change?
			How do the host and microbiome interact to induce host phenotypic plasticity in response to environmental stress changes?
			How does climate change affect the microbiome and host plasticity, particularly regarding environmentally induced dysbiosis (imbalance in the microbial community) and associated functions?
	Population		What are the relationships between the different types and persistence of phenotypic plasticity and generation time, duration of life stages and lifespan and how are they affected under climate change?
			For which traits and taxa can we expect a warming‐induced plasticity squeeze (i.e. a quantitative or qualitative decline in plasticity) or the opening of new plasticity horizons in the form of new quantitative and qualitative plasticity responses?
			What are the consequences of different plasticity responses (e.g. anticipatory, irreversible) for life‐history traits and fitness measures under climate change?
			Will passive plastic responses that are more constrained (e.g. the temperature–size rule) promote or hinder adaptive plasticity to maintain species interactions (e.g. trophic relationships, mutualisms) under climate change?
			Which plastic responses to warming will be maladaptive in the context of species interactions (e.g. lead to greater predation, loss of mutualists etc.)?
		Community/ecosystem	For which traits and plasticity responses do we need a better understanding of the impact of warming on plasticity to make better predictions at the population, community and ecosystem level?
			Which communities and ecosystems will be most affected by the direct and indirect effects of warming on phenotypic plasticity?
			Which communities and ecosystems lend themselves to the simultaneous development of the bottom‐up and top‐down approaches to study the role of warming‐induced changes in phenotypic plasticity?

## V. CONCLUSIONS

(1) It is now well established that phenotypic plasticity underlies crucial climate‐relevant trait differences between individuals and populations. We argue that an integrative study of phenotypic plasticity and its underlying molecular mechanisms will lead to a better understanding of climate‐sensitive variations in individuals, populations, and community dynamics.
(2) In recent years, several studies have focused on the role of genetic diversity as a major determinant of population resilience to environmental change as well as on the distribution of genetic diversity throughout the eukaryotic tree of life (Romiguier *et al*., 2014; De Kort *et al*., 2021). There is now an urgent need for research programmes also to investigate the distribution of climate‐relevant plasticity to identify those taxa that are most sensitive to climate change. Multiple theoretical models, experiments and analyses of empirical data sets have explored the consequences of warming‐induced passive phenotypic plasticity (such as temperature‐dependent feeding and metabolic rates) for the structure and dynamics of populations and communities, while our understanding for the consequences of active plasticity is still fragmentary.
(3) As a way forward, we propose an interdisciplinary framework that combines a more mechanistic, bottom‐up approach with a holistic, top‐down approach (Fig. 2), and highlight outstanding questions for future research (Table 2).
(4) We further advocate for the study of multi‐trait *versus* single‐trait studies, as the former are more informative to understanding individual resilience to the direct and indirect effects of warming.
(5) Finally, classifying plastic traits into those helping to avoid adverse climate conditions and those that support tolerating such conditions will enhance our understanding of how species cope with climate change.

## AUTHOR CONTRIBUTIONS

Coordination of manuscript development: A.St.; conceptualisation: A.St., D.B., P.K., A.Se., C.C.P., E.D., L.D.M., O.E., L.G., J.I.J., C.M., A.N., V.O., J.A.M.R., A.G.R., E.V.V., M.S.; writing – original draft: A.St., D.B., P.K., A.Se., C.C.P., E.D., L.D.M., O.E., L.G., J.I.J., C.L., C.M., A.N., V.O., J.A.M.R., A.R., M.S., E.V.V. Writing – review & editing complete draft: A.St., D.B., P.K., A.Se., L.G., A.N., L.D.M., J.A.M.R. Visualisation: L.G., E.V.V., C.C.P., A.St., D.B.
